# Immune and inflammatory mechanisms in asthma: insights into epigenetic modifications

**DOI:** 10.3389/fimmu.2025.1677552

**Published:** 2025-10-08

**Authors:** Haixia Fan, Bomeng Zhao, Huiyan Niu, Yan Li, Lu Zhai, Limantian Wang, Shudan Deng, Jie Gao, Xiaoling Gao

**Affiliations:** ^1^ First Hospital of Shanxi Medical University, Taiyuan, Shanxi, China; ^2^ Second College of Clinical Medicine, Shanxi Medical University, Taiyuan, Shanxi, China; ^3^ First College of Clinical Medicine Shanxi Medical University, Taiyuan, Shanxi, China; ^4^ The Second Hospital of Shanxi Medical University, Taiyuan, Shanxi, China

**Keywords:** asthma, genetic epigenesis, immunity, DNA methylation, microRNAs, long noncoding RNA, gene-environment interaction, biomarkers

## Abstract

**Background:**

Asthma is a chronic respiratory disease influenced by genetic and environmental factors. Emerging evidence highlights epigenetics as a key regulatory mechanism in asthma development.

**Objective:**

This research aimed to summarize current evidence on immune–epigenetic mechanisms in asthma and to identify global research hotspots through bibliometric analysis.

**Methods:**

A systematic search was conducted in the Web of Science Core Collection(WoSCC) and Scopus databases for studies published between 1980 and July 2025. Following PRISMA guidelines, duplicate removal and quality control were performed. Eligible articles were analyzed using CiteSpace, VOSviewer, and the R bibliometrix package to evaluate publication trends, countries, institutions, authors, journals, co-cited references, and keyword clusters.

**Results:**

A total of 4,020 unique publications were included. By utilizing data from both the WoSCC and Scopus, research output has risen markedly since 2010, with the United States and China leading in productivity and collaboration. Harvard University and the University of California System emerged as central institutions, while influential authors included Ian M. Adcock, Juan C. Celedón, and Peter J. Barnes. Leading journals, like Clinical Epigenetics and the Journal of Allergy and Clinical Immunology, have seen a steady increase in interdisciplinary research contributions over the years. Through keyword clustering, we identified four major research hotspots: immune and inflammatory mechanisms, epigenetic and regulatory mechanisms, environmental exposures and gene–environment interactions, and epigenetic therapies and biomarkers for precision medicine.

**Conclusion:**

Epigenetic research in asthma is rapidly expanding, with increasing international collaboration. Future efforts should focus on translating mechanistic insights into clinical applications by validating biomarkers, refining patient stratification, and advancing epigenetic-based therapeutic strategies.

## 1 Introduction

Asthma is a persistent and complex respiratory condition characterized by airway inflammation, heightened responsiveness, and structural changes. Although corticosteroids and bronchodilators are commonly used to manage symptoms, the disease still affects more than 300 million people worldwide, placing a growing burden on healthcare systems (1, 2). The high prevalence and heterogeneity of the disease underscore the limitations of current symptom-directed therapies and the need to target underlying molecular and immune pathways that drive progression and exacerbations (2). In recent years, increasing attention has been paid to epigenetic mechanisms in asthma (3). These mechanisms act as a critical bridge between environmental exposures and molecular pathways. They also provide new perspectives for understanding disease pathogenesis and identifying potential therapeutic targets. Epigenetic modifications, including DNA methylation, histone modifications, and non-coding RNAs (ncRNAs) such as microRNAs (miRNAs) and long non-coding RNAs (lncRNA) (4), have been shown to regulate immune and inflammatory pathways central to asthma pathogenesis (5, 6). These mechanisms influence cytokine expression and immune cell differentiation, thereby linking epigenetic regulation with airway inflammation (7). Understanding these processes is essential for clarifying asthma heterogeneity and therapeutic strategies. For example, changes in DNA methylation at the T helper type 2 (Th2) locus can promote type 2 inflammation. Dysregulated microRNAs, such as microRNA-155 (miR-155) and microRNA-21 (miR-21), further amplify interleukin-4 (IL-4) and interleukin-13 (IL-13) signaling. In addition, impaired regulatory circuits, including reduced interleukin-10 (IL-10)/Treg function, are linked to abnormal epigenetic control (8). Notably, reductions in histone deacetylases (HDACs)—particularly histone deacetylase 2 (HDAC2)—have been associated with corticosteroid insensitivity in severe asthma, highlighting an epigenetic basis for treatment resistance (9).

Environmental factors, including allergens, air pollutants, infections, and tobacco smoke, can trigger epigenetic changes in both epithelial and immune cells. These changes contribute to Th2-driven inflammation, resistance to corticosteroids, and increased disease severity (10, 11). At the same time, advances in molecular biology have highlighted the therapeutic potential of targeting these epigenetic pathways. Therapies like histone deacetylase inhibitors (HDACis), DNA methylation inhibitors, and miRNA-based treatments are being investigated as precision approaches, providing new possibilities for diagnosis, prognosis, and treatment (8, 12).

While narrative reviews have summarized selected aspects of epigenetics in asthma, and mechanistic studies have focused on isolated pathways, there remains a lack of systematic reviews integrating bibliometric analysis to map the field’s evolution, research hotspots, and global collaborations (13). Addressing this gap is essential to contextualize mechanistic insights, highlight emerging themes, and inform translational opportunities. Therefore, this study conducts a bibliometric analysis of articles regarding the epigenetic mechanisms of immunity and inflammation in asthma. By integrating these perspectives, we aim to provide a structured overview of current knowledge, identify key contributors and collaboration networks, and outline future directions for precision medicine in asthma.

## 2 Methods

### 2.1 Eligibility criteria

Studies that investigate the epigenetic mechanisms (DNA methylation, histone modification, non-coding RNA (ncRNA)) in asthma, involving human or animal models, and report results related to immune or inflammatory pathways will be included. For bibliometric analysis, only articles and reviews will be retained. Exclude non-English papers and studies that do not have original data on epigenetic mechanisms. To minimize the risk of selection bias, two reviewers independently applied the eligibility criteria, with disagreements resolved by discussion.

### 2.2 Search strategy

The WoSCC is known for its rigorous journal selection and reliable citation tracking, effectively capturing the impact and dissemination of scholarly work. Scopus, with its broad disciplinary coverage and advanced citation tools, excels in supporting interdisciplinary research. Combining WoSCC and Scopus provides a more comprehensive and accurate bibliometric analysis, offering deeper insights into research trends and academic developments.

**Figure 1:**
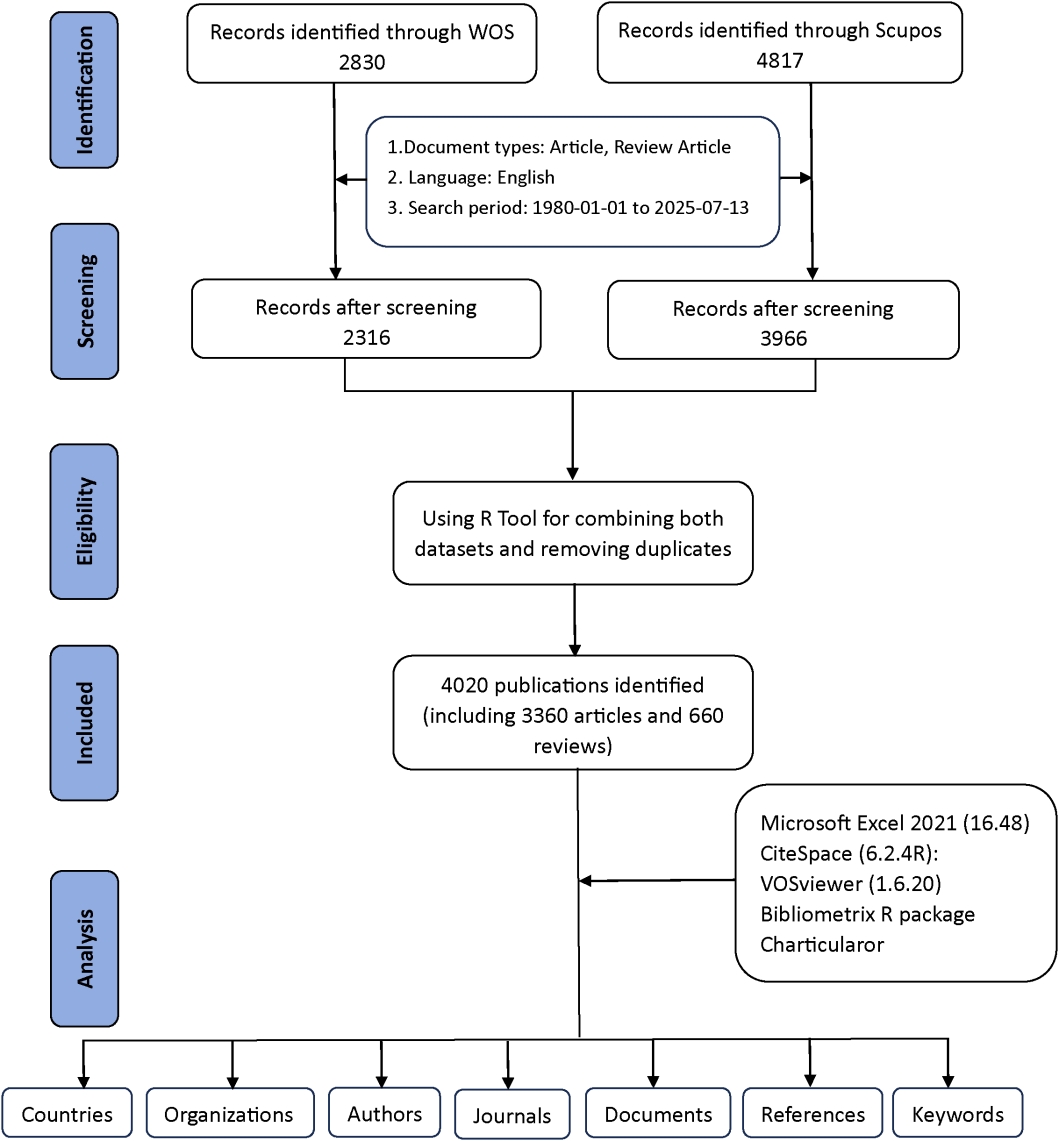
The flowchart for literature search, selection and analysis.

**Figure 1** provides a detailed explanation of the data retrieval and exclusion criteria. Initially, to acquire bibliometric data, search terms were employed in the WoSCC and Scopus databases on July 13, 2025. A preliminary search was subsequently performed based on the publication’s title, abstract, and keywords. For the selection of relevant terms, we reviewed several previous publications in the literature (14, 15). Asthma-related terms= (“asthma” OR “asthmas” OR “asthmatic” OR “bronchial asthma” OR “bronchial asthmas”). Epigenetics-related terms=(“epigenetic*” OR “epigenomic*” OR “DNA methylation” OR “chromatin remodel*” OR “genomic imprinting” OR “methyltransferase*” OR “demethylase*” OR “Hypermethylation” OR “Hypomethylation” OR “CpG island” OR “histone modification*” OR “histone methylation” OR “histone phosphorylation” OR “histone deacetylase*” OR “histone acetyltransferase*” OR “histone glycosylation” OR “histone acetylation” OR “ubiquitination of histone” OR “RNA modification*” OR “RNA methylation” OR “RNA hydroxymethylation” OR “riboswitch” OR “RNA acetylation” OR “non-coding RNA*” OR “noncoding RNA*” OR “long noncoding RNA*” OR “Long Non-Coding RNA*” OR “lncRNA*” OR “long ncRNA*” OR “MicroRNA*” OR “micro RNA*” OR “micro-RNA*” OR “miRNA*” OR “pri-miRNA*” OR “RNA editing” OR “m6A” OR “N6-methyladenosine” OR “5-methylcytosine” OR “circRNA*” OR “Circular RNA*” OR “circ-RNA*” OR “epitranscriptomic*” OR “ATAC-seq” OR “ChIP-seq” OR “bisulfite sequencing” OR “epigenetic therapy*” OR “epigenetic drug*” OR “epigenetic clock”). By combining relevant keywords for preliminary searches, a total of 2,830 documents from WoSCC and 4,817 documents from Scopus were generated.

Exclusions were applied to remove Proceeding Papers, Corrections, Early Access articles, News Items, Book Chapters, Retractions, Reprints, Biographical Items, Book Reviews, Meeting Abstracts, Editorial Materials, and Letters. Only English-language articles and reviews published between 1980 and 2025 were retained. In the end, WoSCC had 2,316 documents remaining, while Scopus had 3,966 documents remaining (**Figure 1**).

Additionally, the two datasets were merged using the bibliometrix R package. Duplicate records were identified primarily through matching of titles, authors, publication years, and DOIs. In cases where DOIs were missing, a combination of title and first author was used to detect duplicates. After automated matching, all potential duplicates were manually checked to ensure accuracy. This procedure avoided double-counting of the same publication indexed in both WoSCC and Scopus. Following this quality-control step, a total of 4,020 unique studies were retained for further analysis, including 3,360 articles and 660 reviews (2,316 from WoSCC and 1,704 from Scopus) (**Figure 1**). These steps were implemented to reduce duplication bias and ensure data accuracy.

### 2.3 Study selection

Two reviewers independently screened titles and abstracts, followed by full-text evaluation against eligibility criteria. Discrepancies were resolved by discussion. After duplicate removal, 4,020 unique studies were included for further analysis.

### 2.4 Data extraction

For each eligible study, data were extracted on publication year, study type, population/sample, investigated epigenetic mechanisms, immune or inflammatory outcomes, and key findings. For the bibliometric component, metadata (e.g., authors, institutions, journals, citations, and keywords) were also collected. To reduce the risk of data extraction bias, two reviewers independently performed the extraction, with cross-checking to ensure consistency.

### 2.5 Bibliometric and scientometric analysis

Bibliometric and scientometric analyses were conducted using multiple complementary tools to ensure robustness. CiteSpace (6.2.4R, 64-bit Advanced Edition) was employed to generate knowledge maps of authors, institutions, and keywords, with a one-year time slice spanning 1980–2025 (16). Author and institution nodes were limited to the top 25 per slice, while keyword networks were pruned with pathfinder and merged techniques to highlight major thematic clusters. VOSviewer (1.6.20) was applied with full counting to construct visual representations of collaborative networks, capturing co-authorship and institutional partnerships (17). The R package *bibliometrix* (https://www.bibliometrix.org) (18), was used for historiographic mapping and calculation of bibliometric indicators, including g-index (19), h-index (20), number of citations (NC), and number of publications (NP) (18), thereby quantifying research productivity and impact. To support data management, Microsoft Excel 2021 was used to organize and clean initial datasets, while the Online Analysis Platform of Literature Metrology (https://bibliometric.com/) provided intuitive visualization of citation dynamics. Together, these tools enabled a comprehensive evaluation of publication trends, influential contributors, collaborative structures, and emerging hotspots in asthma epigenetics research.

## 3 Results

### 3.1 Annual publications trends

**Figure 2:**
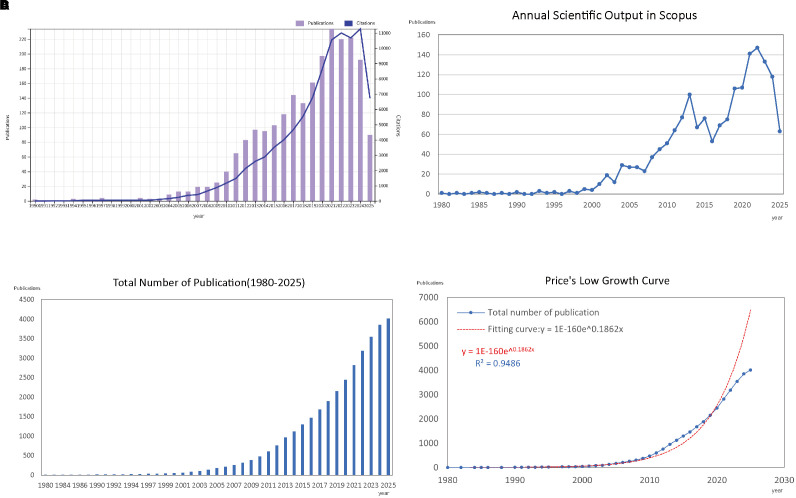
Overview of publications and citations on asthma in epigenetics field (1980–2025). **(A)** Annual trends in the number of publications (bars) and citations (line) retrieved from the WoSCC database. **(B)** Annual trends in publications (bars) and citations (line) retrieved from the Scopus database. **(C)** Cumulative number of publications over time, illustrating the overall growth of the field. **(D)** Price’s Law curve fitting analysis of cumulative publications, demonstrating the exponential growth pattern of asthma–epigenetics research.

Our bibliometric analysis, covering the period from 1980 to 2025, showed a significant increase in both the number of publications and citations in the field of asthma and epigenetics. We retrieved 4,020 documents from WoSCC and Scopus, with an annual growth rate of 11.98%, reflecting a steadily expanding research landscape over the past four decades (**Supplementary Table S1**). As illustrated in **Figure 2A**, both annual publications and citations saw steady growth in the early years, but from the early 2000s onward, there was a noticeable increase, with a sharp rise occurring after 2010. **Figure 2B** displays the trend from the Scopus database, which aligns with those shown in **Figure 2A**. **Figure 2C** depicts the cumulative number of publications over time, highlighting a clear upward trajectory, especially in the last 15 years. To further analyze this growth, **Figure 2D** presents a Price’s Law growth curve fitting analysis. The model fitting equation, y = 1E-160e^0.1862x^, with an R² value of 0.9486, shows a strong alignment with the observed data, confirming an exponential growth pattern consistent with Price’s Law of scientific literature development. This indicates that the field has not only expanded in volume but also exhibits the characteristics of a rapidly evolving scientific discipline.

### 3.2 Distributions of countries/regions

**Figure 3:**
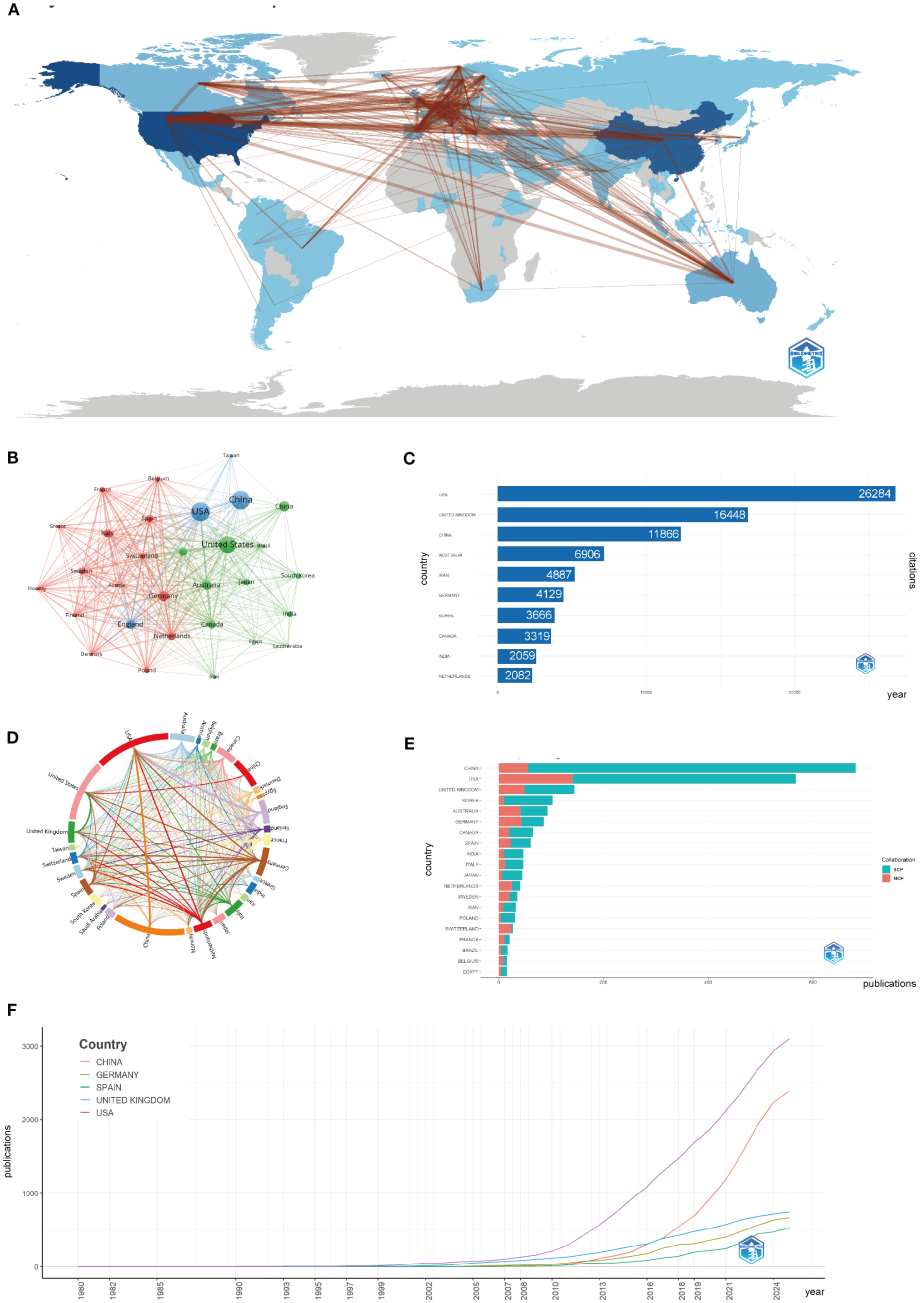
Global trends and collaboration networks in scientific publications on asthma in epigenetics field (1980–2025). **(A)** Country/Region Collaboration Map. Darker shades of blue indicate higher collaboration rates, and the width of the link lines represents the strength of collaboration between two countries. **(B)** Country Clustering Analysis(network). Each node represents a country/region. Node size is proportional to publication output. Edge thickness indicates co-authorship link strength between countries. Node colors denote collaboration clusters detected by the layout algorithm; countries in the same color cluster collaborate more frequently with one another. Spatial proximity reflects higher relatedness (stronger links). **(C)** Top 10 Countries/Regions by Citation Impact. Horizontal bars show total citation counts for publications affiliated with each country/region (labels on bars give counts). **(D)** Network Map of National Research Output and Cooperative Relations (chord diagram). Each outer arc corresponds to a country/region; arc length reflects that country’s overall collaborative output within the network. Ribbons connect pairs of countries; ribbon width represents the strength of their collaboration (number of co-authored documents). Ribbon colors follow the color of the originating arc. **(E)** Leading countries by number of studies and collaboration type. Stacked horizontal bars display each country’s publication count, partitioned into single-country publications (SCP) and multiple-country publications (MCP). Teal segments denote SCP and salmon/red segments denote MCP (legend “Collaboration” in the panel). The MCP proportion indicates the extent of international collaboration. **(F)** Top 5 Countries/Regions by Publications. Lines show the cumulative number of publications from 1980 to 2025. The x-axis is year; the y-axis is cumulative document count.

**Figures 3A, B, D** display the global research network, with darker blue shades representing stronger collaborations. The USA, China, and the United Kingdom show the most intense collaborative connections, with dominant networks linking North America, Europe, and East Asia. In contrast, regions such as Africa and Latin America experience fewer collaborations, revealing a geographical imbalance in global scientific partnerships.

The growth of global research became especially apparent after 2000, with China and the USA leading in both the number of publications and citation impact. Germany, Spain, and the UK have shown steady growth, while China’s recent surge is driven by increased funding and infrastructure investments in asthma epigenetics research. **Figures 3C, E** highlight this rapid expansion, with both China and the USA maintaining strong publication output. China’s post-2010 rise in publications is especially pronounced, marking a shift towards more specialized topics, such as gene-environment interactions in asthma. Finally, in **Supplementary Table S2**; **Figure 3F** show China’s rise as a dominant force in the field, with a significant increase in publications after 2010. The USA and the United Kingdom are at the forefront of research output.

### 3.3 Distribution by institutions

In addition to identifying leading institutions, our analysis explores their research productivity and collaboration patterns in asthma epigenetics.

**Figure 4:**
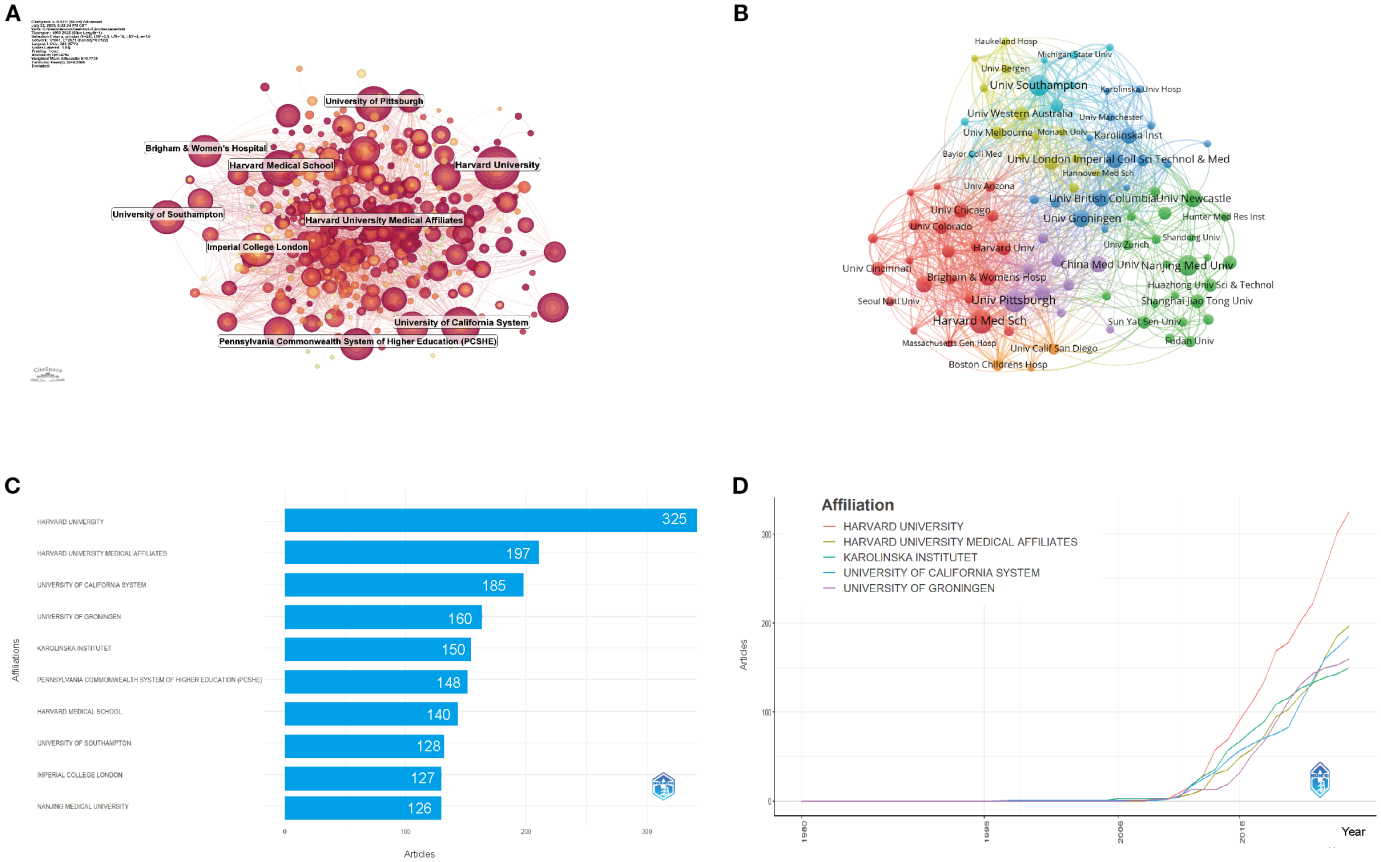
Institutional contributions and collaboration networks on asthma in epigenetics field (1980–2025). **(A)** Research institution network. Nodes represent institutions, with size reflecting publication volume and line thickness indicating collaboration strength. **(B)** Institutional collaboration network. Node size corresponds to the extent of collaboration activity. Distinct colors denote clusters, generally reflecting regional or institutional groupings. Line thickness indicates the intensity of cooperation based on the number of shared publications. **(C)** Top 10 Institutions by Citation Impact. **(D)** Institutional publication trends. The x-axis denotes years, and the y-axis indicates publication counts.

**Figure 4A** shows the institutional co-occurrence network, revealing a complex web of collaborations. Harvard University emerges as a central hub, linking institutions across North America, Europe, and Asia. Its strong connections with Harvard Medical Affiliates, Brigham and Women’s Hospital, and the University of Pittsburgh create a core cluster of knowledge exchange at the heart of the field. Other significant clusters are centered around the University of California System, Imperial College London, and Nanjing Medical University, indicating geographically dispersed but thematically aligned research centers. These institutions serve as crucial nodes in global collaborations, driving interdisciplinary progress. **Figure 4B** highlights institutions like the University of Pittsburgh, University of Southampton, and Pennsylvania Commonwealth System of Higher Education (PCSHE) as vital bridges connecting North American and European research networks. Their presence in dense collaborative clusters emphasizes their role in fostering cross-regional knowledge exchange. This growing trend toward inter-institutional collaboration reflects the inherently multidisciplinary nature of asthma epigenetics, which spans genomics, immunology, environmental science, and data analytics. As a result, multi-institutional partnerships have become critical for boosting research impact, fostering methodological diversity, and enhancing global visibility.

**Figures 4C**, **D** reveal that from 1980 to 2025, the top five institutions by total output were: Harvard University (325 publications, 8.09%), Harvard Medical Affiliates (197, 4.90%), University of California System (185, 4.60%), University of Groningen (160, 3.98%), and Karolinska Institute (150, 3.73%). These institutions not only show consistent productivity but also early involvement in the field, with Harvard leading in both publication volume and initial contributions since the mid-2010s.

### 3.4 Distributions of authors and co-cited authors

The scholarly landscape of asthma and epigenetics research from 1980 to 2025 is characterized by extensive collaboration. A total of 20,197 authors contributed to 4,020 publications, with an average of 7.14 co-authors per paper, reflecting the multidisciplinary nature of the field (**Supplementary Table S1**). Single-authored works were rare, accounting for only about 1% of all publications. Moreover, international collaborations represented 14.73% of the total, showing a steady increase in cross-border partnerships, particularly among institutions in Europe, North America, and East Asia (**Supplementary Table S1**).

**Figure 5:**
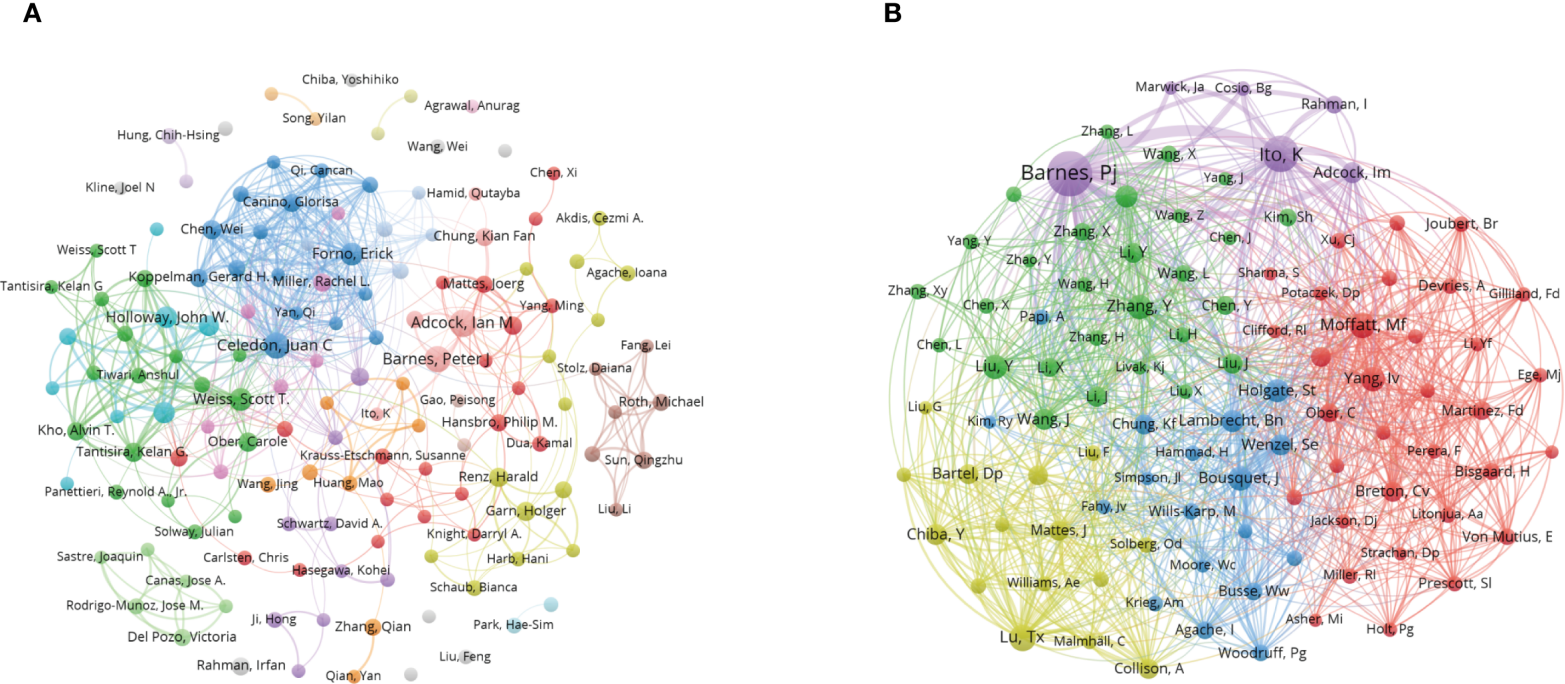
Co-authorship and co-cited authorship networks on asthma in epigenetics field (1980–2025). **(A)** Co-authorship network: Nodes represent authors, sized by publication count. Colors indicate collaboration clusters. **(B)** Co-cited authorship network: Nodes, connected by collaborative publications, have edge thickness proportional to joint works. Clusters, marked by colors, reveal research groups.

**Figures 5A**, **B** both highlight patterns of collaboration and influence among key authors. **Table 1** quantifies author influence, showcasing metrics like the h-index (20), g-index (19), and total citation counts (TC). The h-index is a widely used bibliometric measure that tracks the number of researcher’s papers cited at least h times, reflecting consistent productivity. In contrast, the g-index emphasizes highly cited papers, highlighting significant impact in specific research areas. For instance, Ian M. Adcock has an h-index of 22 and a g-index of 26, demonstrating his strong contribution to the field. On the other hand, Juan C. Celedón, with an h-index of 20 and a g-index of 40, shows a greater concentration of influential works, reflecting his broader citation impact. **Figure 5B** displays the co-cited author network, showcasing the collective influence of leading figures in asthma epigenetics, including authors such as Ian M. Adcock, Juan C. Celedón, and John W. Holloway. These individuals act as central nodes in the network, linked by a dense web of co-citations. This representation emphasizes collaborative relationships among key contributors, illustrating the strong scholarly connections that drive new insights and discoveries in the field.

**Table 1:** Top 10 authors on asthma in epigenetics field research (1980–2025).

Rank	Author	h_index	g_index	m_index	TC	NP	PY_start	Articles	Articles fractionalized
1	ADCOCK IAN M.	22	26	1.158	1893	26	2007	26	5.40
2	CELEDON JUAN C.	20	40	1.176	1616	46	2009	46	5.41
3	HOLLOWAY JOHN W.	20	35	1.111	1253	36	2008	36	4.72
4	FORNO ERICK	19	39	1.462	1809	39	2013	39	6.08
5	KARMAUS WILFRIED	19	28	1.118	1574	28	2009	28	3.34
6	MELEN ERIK	19	28	1.357	2125	28	2012	28	2.76
7	RENZ HARALD	19	23	1.188	1746	23	2010	23	3.83
8	XU CHENG-JIAN	19	25	1.9	1976	25	2016	25	1.56
9	GARN HOLGER	18	21	1.2	1126	21	2011	21	2.73
10	KOPPELMAN GERARD H.	18	28	1.2	1929	28	2011	28	2.81

TC, Total Citations; NP, Number of Publications; PY_start, Year of First Publication.

### 3.5 Journals and co-journals

**Figure 6:**
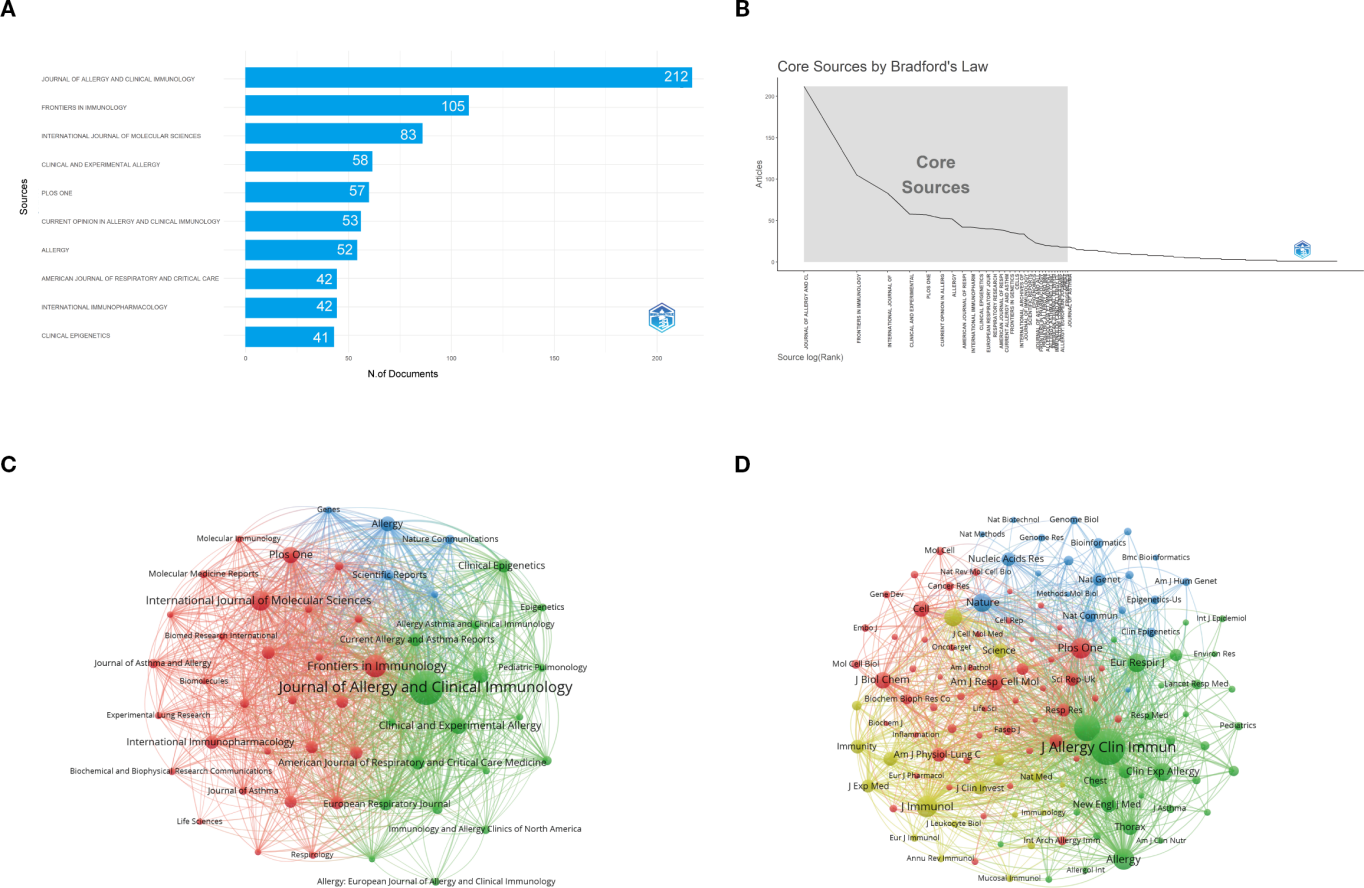
Comprehensive analysis of journals and citation networks related to asthma in epigenetics field (1980–2025). **(A)** Top journals by publication count: X-axis shows article numbers; y-axis lists journals. **(B)** Bradford’s Law analysis: Identifies core journals based on publication distribution. **(C)** Journal citation network. Each node represents a journal. Node size is proportional to the number of publications, while links indicate citation relationships between journals. Line thickness reflects the strength of citation connections. Distinct colors (green, blue, red) denote clusters of journals that frequently cite each other, corresponding to thematic areas of research. **(D)** Journal co-citation network: Each node represents a journal, with size determined by co-citation frequency. Links indicate co-citation ties, with thicker lines reflecting stronger co-citation strength. Colors identify clusters of journals that are commonly co-cited.

**Figure 6A** shows that the *Journal of Allergy and Clinical Immunology* is the most influential source in asthma epigenetics, with 212 publications, highlighting its central role in disseminating high-impact research. It is followed by *Frontiers in Immunology* (105 articles) and the *International Journal of Molecular Sciences* (83 articles), both of which also contribute significantly to shaping the field. **Table 2** further confirms their academic impact: *Journal of Allergy and Clinical Immunology* holds an h-index of 81, a Q1 JCR ranking, and a high citation count, while the other two journals are also Q1-ranked and frequently publish on key topics such as DNA methylation, microRNA regulation, and airway inflammation. These journals’ prominence is reflected not only in output volume but also in academic visibility and reputation. Most are Q1 journals according to JCR, indicating their high impact and wide recognition. **Figure 6B**, consistent with Bradford’s Law, reveals a skewed distribution of publications, where a small number of core journals—such as *Journal of Allergy and Clinical Immunology*, *PLOS ONE*, and *Allergy*—account for a majority of the research output. **Figure 6C** illustrates the cumulative growth of publications over time, further confirming that leading journals, like those shown in **Figure 6A**, exhibit a continuous increase in output, indicating their lasting impact and growing involvement in the field of asthma epigenetics. **Figure 6D** visualizes the journal co-citation network, revealing clusters of frequently co-cited journals, which reflect strong thematic communities within the field—particularly around immunology, molecular biology, and respiratory disease.

**Table 2:** Top 10 influential journals on asthma in epigenetics field research (1980–2025).

Rank	Source	h_index	g_index	m_index	TC	NP	PY_start	IF	JCR
1	JOURNAL OF ALLERGY AND CLINICAL IMMUNOLOGY	81	125	2.613	17459	212	1995	11.2	Q1
2	FRONTIERS IN IMMUNOLOGY	34	59	3.091	3836	105	2015	5.9	Q1
3	INTERNATIONAL JOURNAL OF MOLECULAR SCIENCES	31	52	2.214	2859	83	2012	4.9	Q1
4	ALLERGY	30	51	1.364	2699	52	2004	12	Q1
5	AMERICAN JOURNAL OF RESPIRATORY AND CRITICAL CARE MEDICINE	30	42	1.25	4627	42	2002	19.4	Q1
6	CLINICAL AND EXPERIMENTAL ALLERGY	29	50	0.879	2613	58	1993	5.2	Q1
7	CURRENT OPINION IN ALLERGY AND CLINICAL IMMUNOLOGY	28	40	1.167	1731	53	2002	2.6	Q2
8	JOURNAL OF IMMUNOLOGY	27	34	1	3307	34	1999	3.4	Q2
9	PLOS ONE	27	47	1.588	2261	57	2009	2.6	Q2
10	EUROPEAN RESPIRATORY JOURNAL	26	40	1.182	5534	40	2004	21	Q1

IF, Impact Factor; JCR, Journal Citation Reports.

### 3.6 References and articles

The field of asthma epigenetics is marked by significant contributions from key authors, influential journals, and widely cited publications. The following analysis delves into key insights from **Supplementary Table S1**. The dataset includes 4,020 documents with 221,863 references, reflecting the extensive body of work in asthma epigenetics between 1980 and 2025. On average, documents are 7.54 years old and receive 53.47 citations each, demonstrating the substantial impact these studies have on the academic community.

In **Table 3**, the most locally cited publications are led by Lu TX (2009) (21) and Mattes J (2009) (22), with 146 and 146 local citations, respectively. These groundbreaking studies have played a key role in shaping asthma epigenetics research, especially in the area of miRNA regulation. For instance, Lu TX’s work on miR-21 and its role in allergic airway inflammation is crucial for understanding the molecular mechanisms behind asthma. Likewise, Mattes J’s research on microRNA-126(miR-126) offers valuable insights into how T-helper cells are regulated in allergic diseases, laying the groundwork for our understanding of asthma’s molecular basis.

**Table 3:** Top 25 most local cited publications based on bibliometrix analysis (1980–2025).

Rank	First author	Year	Journal	Paper	DOI	Local citations	Global citations	LC/GC ratio (%)	Normalized local citations	Normalized global citations
1	LU TX	2009	J IMMUNOL	MicroRNA-21 Is Up-Regulated in Allergic Airway Inflammation and Regulates IL-12p35 Expression	10.4049/jimmunol.0803560	146	532	27.44	13.32	4.31
2	MATTES J	2009	P NATL ACAD SCI USA	Antagonism of microRNA-126 suppresses the effector function of TH2 cells and the development of allergic airways disease	10.1073/pnas.0905063106	146	374	39.04	13.32	3.03
3	SOLBERG OD	2012	AM J RESP CRIT CARE	Airway epithelial miRNA expression is altered in asthma	10.1164/rccm.201201-0027OC	119	198	60.10	14.60	1.94
4	LEVÄNEN B	2013	J ALLERGY CLIN IMMUN	Altered microRNA profiles in bronchoalveolar lavage fluid exosomes in asthmatic patients	10.1016/j.jaci.2012.11.039	99	257	38.52	20.44	3.11
5	YANG IV	2015	J ALLERGY CLIN IMMUN	DNA methylation and childhood asthma in the inner city	10.1016/j.jaci.2015.01.025	96	180	53.33	15.87	2.84
6	WILLIAMS AE	2009	PLOS ONE	MicroRNA expression profiling in mild asthmatic human airways and effect of corticosteroid therapy	10.1371/journal.pone.0005889	95	167	56.89	8.67	1.35
7	KUMAR M	2011	J ALLERGY CLIN IMMUN	Let-7 microRNA-mediated regulation of IL-13 and allergic airway inflammation	10.1016/j.jaci.2011.04.034	95	261	36.40	13.54	3.02
8	COLLISON A	2011	J ALLERGY CLIN IMMUN	Inhibition of house dust mite-induced allergic airways disease by antagonism of microRNA-145 is comparable to glucocorticoid treatment	10.1016/j.jaci.2011.04.005	92	189	48.68	13.11	2.19
9	MALMHÄLL C	2014	J ALLERGY CLIN IMMUN	MicroRNA-155 is essential for T(H)2-mediated allergen-induced eosinophilic inflammation in the lung	10.1016/j.jaci.2013.11.008	92	189	48.68	12.25	2.07
10	XU CJ	2018	LANCET RESP MED	DNA methylation in childhood asthma: an epigenome-wide meta-analysis	10.1016/S2213-2600(18)30052-3	89	166	53.61	17.20	2.34
11	ITO K	2002	AM J RESP CRIT CARE	Expression and activity of histone deacetylases in human asthmatic airways	10.1164/rccm.2110060	84	249	33.73	12.57	2.75
12	MAES T	2016	J ALLERGY CLIN IMMUN	Asthma inflammatory phenotypes show differential microRNA expression in sputum	10.1016/j.jaci.2016.02.018	83	157	52.87	12.11	1.87
13	PERERA F	2009	PLOS ONE	Relation of DNA methylation of 5'-CpG island of ACSL3 to transplacental exposure to airborne polycyclic aromatic hydrocarbons and childhood asthma	10.1371/journal.pone.0004488	80	321	24.92	7.30	2.60
14	REESE SE	2019	J ALLERGY CLIN IMMUN	Epigenome-wide meta-analysis of DNA methylation and childhood asthma	10.1016/j.jaci.2018.11.043	80	150	53.33	13.65	3.20
15	PERRY MM	2014	AM J RESP CELL MOL	Airway smooth muscle hyperproliferation is regulated by microRNA-221 in severe asthma	10.1165/rcmb.2013-0067OC	79	135	58.52	10.52	1.48
16	POLIKEPAHAD S	2010	J BIOL CHEM	Proinflammatory role for let-7 microRNAS in experimental asthma	10.1074/jbc.M110.145698	78	174	44.83	12.72	1.02
17	PANGANIBAN RP	2016	J ALLERGY CLIN IMMUN	Circulating microRNAs as biomarkers in patients with allergic rhinitis and asthma	10.1016/j.jaci.2016.01.029	78	176	44.32	11.38	2.10
18	ITO K	2005	NEW ENGL J MED	Decreased histone deacetylase activity in chronic obstructive pulmonary disease	10.1056/NEJMoa041892	74	755	9.80	16.00	10.20
19	HOLLINGSWORTH JW	2008	J CLIN INVEST	*In utero* supplementation with methyl donors enhances allergic airway disease in mice	10.1172/JCI34378	74	417	17.75	11.29	4.10
20	LU TX	2011	J IMMUNOL	MicroRNA-21 limits *in vivo* immune response-mediated activation of the IL-12/IFN-gamma pathway, Th1 polarization, and the severity of delayed-type hypersensitivity	10.4049/jimmunol.1101235	74	308	24.03	10.55	3.57
21	TSITSIOU E	2012	J ALLERGY CLIN IMMUN	Transcriptome analysis shows activation of circulating CD8+ T cells in patients with severe asthma	10.1016/j.jaci.2011.08.011	73	163	44.79	8.96	1.60
22	FORNO E	2019	LANCET RESP MED	DNA methylation in nasal epithelium, atopy, and atopic asthma in children: a genome-wide study	10.1016/S2213-2600(18)30466-1	73	146	50.00	12.45	3.11
23	JARDIM MJ	2012	AM J RESP CELL MOL	Distinct microRNA expression in human airway cells of asthmatic donors identifies a novel asthma-associated gene	10.1165/rcmb.2011-0160OC	71	107	66.36	8.71	1.05
24	COLLISON A	2011	BMC PULM MED	Altered expression of microRNA in the airway wall in chronic asthma: miR-126 as a potential therapeutic target	10.1186/1471-2466-11-29	68	121	56.20	9.69	1.40
25	SIMPSON LJ	2014	NAT IMMUNOL	A microRNA upregulated in asthma airway T cells promotes TH2 cytokine production	10.1038/ni.3026	68	200	34.00	9.05	2.19

DOI, Digital Object Identifier.

**Figure 7:**
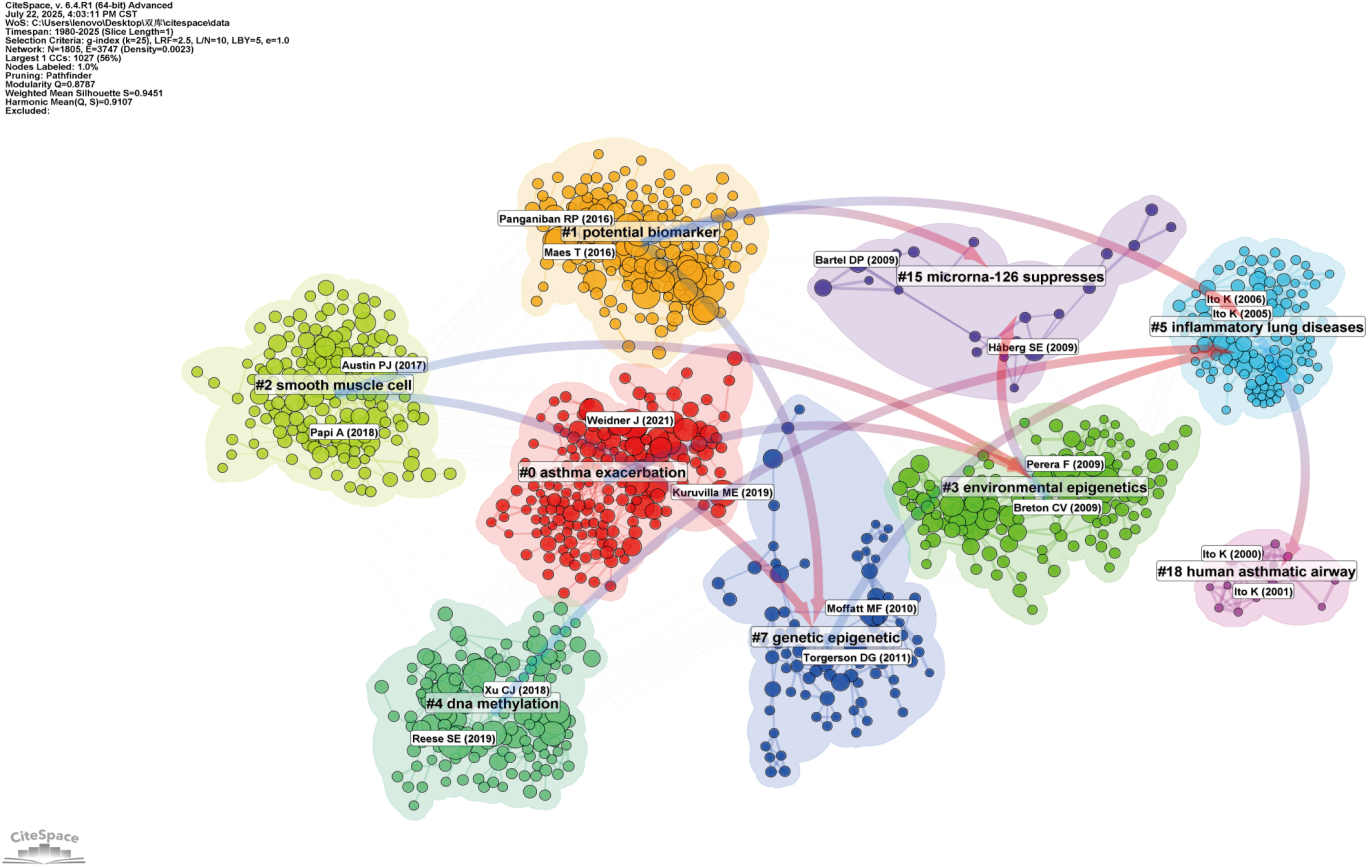
Network visualization of references on asthma in epigenetics field (1980–2025). Clustered co-citation network based on keywords: Nodes, sized by citation frequency, are grouped by modularity with keyword labels for major clusters. Edge thickness reflects co-citation strength; colors highlight thematic diversity.

**Figure 7** displays a co-citation clustering map that reveals the thematic structure of asthma research linked to epigenetics. Several clusters stand out as central to the field, showing a strong focus of scholarly attention and interconnection. Most notably, Cluster 4 (“DNA methylation”) and Cluster 6 (“Epigenetic regulation”) form the core of the map, highlighting the dominant role of epigenetic mechanisms—especially DNA methylation—in asthma research. These clusters demonstrate how modifications in gene expression, without altering the DNA sequence, influence asthma susceptibility and progression. Cluster 3 (“Environmental epigenetics”) emphasizes the connection between environmental factors and epigenetic mechanisms, underscoring the growing importance of pollutants, allergens, and early-life exposures in asthma.

In contrast, Cluster 15 (“MicroRNA-126 suppresses”) represents a focused area, focusing on the regulatory roles of ncRNAs, particularly miRNAs, in asthma-related inflammation. While smaller, this cluster reflects the increasing interest in identifying molecular targets for new therapeutic interventions. Other clusters, like Cluster 0 (“Asthma exacerbation”) and Cluster 1 (“Potential biomarkers”), illustrate broader applications of epigenetic research, including translational studies and the search for biomarkers to advance personalized medicine. These areas, although not entirely focused on epigenetics, highlight the significant clinical impact of epigenetic discoveries on asthma treatment and outcomes.

**Figure 8:**
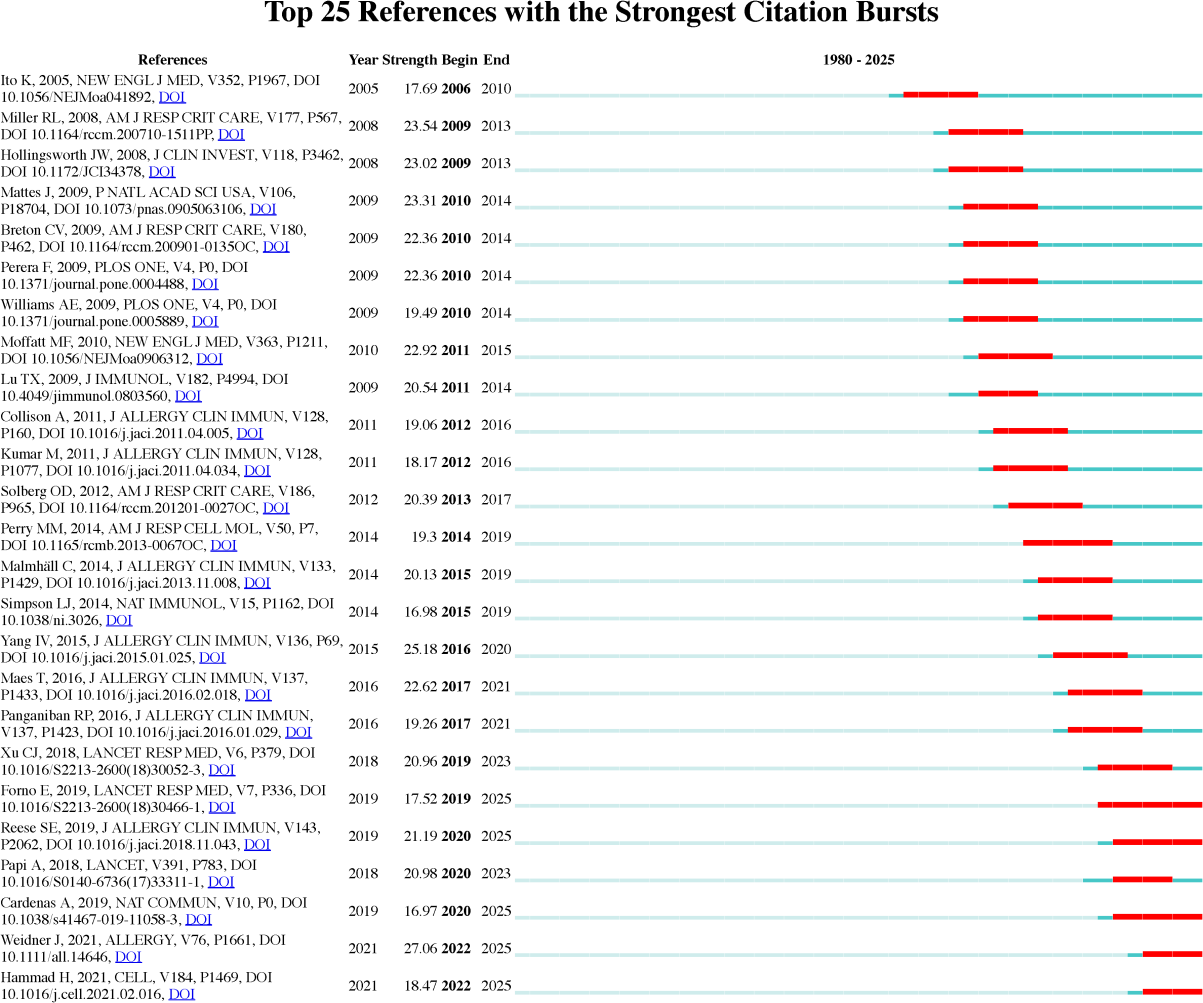
Top 25 references with strongest citation bursts on asthma in epigenetics field. The burst timeline shows the periods during which these bursts occurred, with red segments indicating the time intervals when each reference received heightened attention.

**Figure 8** presents the top 25 references in the field, ranked by their citation burst strength between 1980 and 2025. Citation burst strength measures the intensity of citation frequency over time, revealing periods of high research activity. For instance, Weidner J et al. (2021) (23) stands out with a citation burst strength of 27.06 in 2025, indicating its significant influence on subsequent asthma research. Additional studies, such as those by Hammad H et al. (2021) (24) and Cardenas A et al. (2019) (25), show citation bursts in 2022 and 2020, respectively, reflecting ongoing developments in asthma research and increased interest in epigenetic mechanisms. These citation bursts highlight emerging research trends, particularly in the genetic and environmental factors influencing asthma, marking important indicators of rapidly evolving research fronts in the field.

### 3.7 Keywords analysis

The keyword analysis highlights key trends and focal areas in asthma research related to epigenetics. A total of 21,791 Keywords Plus and 6,909 author keywords were identified, greatly improving the indexing and retrieval of pertinent scholarly content (**Supplementary Table S1**). This extensive dataset provides a deeper insight into emerging topics and research directions, especially in the area of epigenetic mechanisms in asthma.

**Figure 9:**
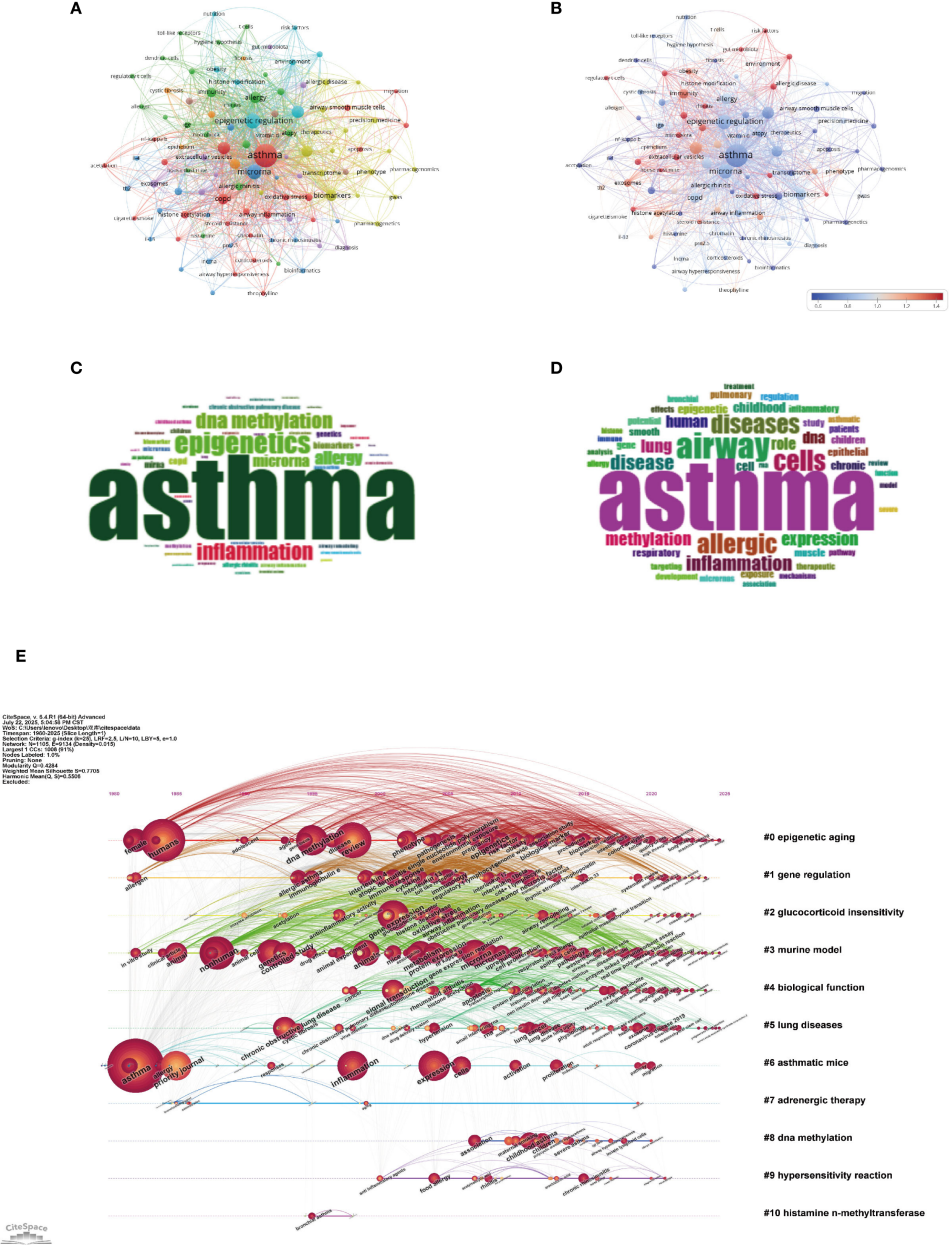
Keywords mapping of asthma in epigenetics field. **(A)** Keywords co-occurrence network: Each node represents a keyword, with node size proportional to its occurrence frequency. Edges indicate co-occurrence relationships, with line thickness reflecting co-occurrence strength. Distinct colors represent thematic clusters identified by modularity analysis. **(B)** Keyword centrality network. Nodes represent keywords, with node size proportional to their occurrence frequency and node color indicating betweenness centrality (redder nodes = higher centrality). Edges denote co-occurrence relationships. This visualization highlights pivotal keywords that bridge multiple clusters. **(C)** Author keywords word cloud: Word size reflects frequency, highlighting prevalent author-assigned terms. **(D)** Title keywords word cloud: Word size indicates frequency, emphasizing key title-derived themes. **(E)** Keyword timeline visualization. The horizontal axis denotes publication years. Each horizontal line corresponds to a thematic cluster, labeled with a representative keyword or phrase. Nodes represent keywords within clusters, sized by occurrence frequency and connected by co-occurrence links. Node positions along the timeline reflect the first year in which the keyword appeared, allowing visualization of topic emergence, persistence, and evolution across time.

As shown in **Figures 9A–D**, the central theme in asthma research remains “asthma” (1601 occurrences, total link strength = 2866), reflecting its dominant role in the field. However, there is an increasing focus on molecular mechanisms, as indicated by keywords such as “epigenetic regulation” (451 occurrences, total link strength = 1036) and “microRNA” (421 occurrences, total link strength = 931). These trends signal a shift towards understanding the epigenetic factors underlying asthma pathogenesis, especially with the growing importance of miRNAs and their role in regulating gene expression in asthma development (26). “Inflammation” (271 occurrences, total link strength = 575) remains a key focus, underscoring the ongoing research into inflammatory pathways in asthma (27). This is further emphasized by the keyword “DNA methylation” (264 occurrences, total link strength = 533), which highlights the importance of epigenetic modifications in immune responses and asthma exacerbations (28). The term “allergy” (207 occurrences, total link strength = 572) also continues to be highly relevant, indicating the close relationship between asthma and allergic conditions (29). The research scope is also expanding beyond asthma-specific mechanisms. Keywords such as “COPD” (185 occurrences, total link strength = 442) and “biomarkers” (191 occurrences, total link strength = 448) indicate increasing interest in chronic obstructive pulmonary disease and its overlap with asthma, particularly in shared inflammatory pathways (30). These findings suggest the potential for cross-disease therapeutic approaches. Additionally, “gene expression” (200 occurrences, total link strength = 558) and “cytokine” (76 occurrences, total link strength = 168) reflect the ongoing exploration of genetic and molecular markers that contribute to asthma (31). Other notable keywords, such as “lung disease” (153 occurrences, total link strength = 342) and “immunity” (141 occurrences, total link strength = 307), highlight the broadening of research to include more general respiratory diseases and immune system contributions to asthma pathogenesis (32, 33). The presence of keywords like “exosomes” (75 occurrences, total link strength = 174) and “histone acetylation” (71 occurrences, total link strength = 172) suggests a growing interest in novel molecular mechanisms and their potential therapeutic implications (34, 35).

In summary, the evolving focus on epigenetics, inflammatory pathways, and cross-disease comparisons highlights a more comprehensive understanding of asthma, paving the way for more targeted and integrated treatments (35–37).

As shown in the keyword timeline (**Figure 9E**) created with CiteSpace, the evolution of asthma research reflects clear thematic and temporal progression from the late 1980s to 2025. Early on, “epigenetic aging” (Cluster 0) became a central focus, gaining increasing relevance through the 2000s and 2010s. This trend highlights the growing awareness of how age-related epigenetic changes contribute to asthma development, potentially linking the aging immune system to chronic respiratory issues (38–40). Subsequently, the rise of keywords like “gene regulation” (Cluster 1) and “glucocorticoid insensitivity” (Cluster 2) from the mid-1990s to early 2000s indicates growing focus on the molecular mechanisms of asthma, particularly regarding steroid-resistant phenotypes. The connection of these terms with clinical challenges in asthma treatment underscores the need for a deeper understanding of therapeutic resistance, particularly in relation to corticosteroid efficacy (35, 41, 42). Over the past decade, there has been a distinct shift towards innovative therapies and the exploration of epigenetic mechanisms. Terms like “adrenergic therapy” (Cluster 7) and “DNA methylation” (Cluster 8) have become increasingly prominent, reflecting the rising importance of precision medicine approaches. The growing attention on “DNA methylation” emphasizes its crucial role in regulating gene expression, which influences immune responses and airway remodeling. As a result, it has emerged as both a key biomarker and a potential target for therapeutic development (28, 35, 38). Recent discussions around topics like “hypersensitivity reaction” (Cluster 9) and “histamine N-methyltransferase” (Cluster 10) highlight the growing recognition of immune dysregulation and histamine metabolism in the worsening of asthma symptoms (43, 44). These developments point to a renewed focus on traditional immunological pathways, now being explored with the tools of modern molecular biology.

The occurrence and link strength values mentioned above are derived from our analysis based on CiteSpace. Collectively, the growing incorporation of epigenetics, systems biology, and therapeutic innovations highlights both a deeper understanding of asthma’s mechanisms and a shift toward personalized treatment strategies (35, 45). As the field progresses, these insights will play a crucial role in improving diagnostic tools and creating targeted therapies that tackle the molecular complexity of asthma’s heterogeneity (46).

## 4 Discussion

The growing global impact of asthma continues to challenge healthcare systems. Although current treatments provide symptom relief, they do not address the underlying mechanisms of the disease. Epigenetic changes—particularly DNA methylation and miRNA regulation, are gaining recognition for their role in immune response and airway inflammation. Our scientometric analysis shows a significant rise in asthma epigenetics research since 2000, driven by advances in technology and increased global collaboration, especially among the U.S., China, and European countries. Emerging areas such as gene-environmental interactions and environmental epigenetics suggest a shift towards more mechanistic and translational research. These developments underscore the potential of epigenetic insights to drive precision medicine and personalized asthma treatments.

### 4.1 Research highlights

The keyword and reference clustering analysis reveals the key areas that researchers are focusing on. Building on these insights, we summarize several prominent research hotspots, including immune and inflammatory mechanisms, epigenetic and regulatory mechanisms, environmental exposures and risk factors, epigenetic therapies and translational applications in asthma, and biomarkers in precision medicine.

#### 4.1.1 Immune and inflammatory mechanisms

Asthma is strongly associated with immune and inflammatory processes that drive its development. One key factor, Ficolin-A (FCN-A), has been highlighted. In asthmatic models, lower serum levels of FCN-A are linked to higher Immunoglobulin E (IgE) production and increased type 2 cytokine expression. This exacerbates asthma symptoms and suggests that FCN-A may help suppress type 2 inflammation in allergic asthma (47). Additionally, the Th2-dominated immune response and Toll-like receptors (TLRs) play central roles in asthma’s pathophysiology, promoting airway hyperresponsiveness and remodeling (48). Genetically predicted levels of macrophage colony-stimulating factor (M-CSF) are associated with a higher risk of developing asthma. This finding indicates a direct link between macrophage activity and asthma susceptibility (48).

Several cytokines, including IL-4, IL-10, and IL-13, are crucial to the inflammation and immune responses in asthma. IL-4 is central to Th2-polarized allergic airway inflammation. Elevated IL-4 levels have been observed in asthma, including in FCN-A knockout mice, underscoring its role in symptom worsening (47). Treatments such as Stimulator of Interferon Genes (STING) inhibition and CA-NPs-Quercetin can lower IL-4 expression, indicating its potential as a therapeutic target to manage asthma-related inflammation (49, 50). Additionally, IL-4 is involved in fibroblast reprogramming, which is crucial for driving Th2 responses in asthma (51). Notably, the relationship between IL-4 and IL-13 is context-dependent: while IL-4 usually promotes Th2-driven inflammation, in group 2 innate lymphoid cells (ILC2s) it may suppress IL-13 expression, suggesting a cell-specific regulatory mechanism (52). IL-13 itself is a major contributor to airway inflammation and hyperresponsiveness. Its levels are elevated in several asthma models, including ovalbumin (OVA)-induced asthma and house dust mite (HDM)-induced asthma, confirming its role in the inflammatory process (53, 54). Treatments that reduce IL-13 expression, like Q/SC extract, suggest that targeting IL-13 can modulate allergic airway inflammation (55). IL-10 is another important cytokine that helps regulate inflammation in asthma. Higher IL-10 levels have been linked to better asthma control and less airway inflammation, as observed with treatments like CA-NPs-Quercetin and Angiotensin- (1–7)(Ang- (1–7)) (50, 56). Conversely, reduced IL-10 levels are linked to unfavorable outcomes in pediatric asthma patients, indicating its importance in prognosis (57). IL-4, IL-10, and IL-13 jointly shape asthma inflammation and represent potential therapeutic targets. Landmark studies have shown that DNA methylation at Cytosine–phosphate–Guanine dinucleotide (CpG) sites in immune-related genes (e.g., IL-4, IL-13, Forkhead box P3(FOXP3)) is linked to Th2/T helper 17 cells (Th17) polarization and asthma severity. This suggests a direct regulatory influence on immune cell differentiation and function (58, 59). These findings strengthen the idea that the epigenome plays a key role in linking environmental exposures to immune dysregulation in asthma. Immune–epigenetic interactions drive asthma pathogenesis and point to new therapeutic directions.

#### 4.1.2 Epigenetic and regulatory mechanisms

Researchers have revealed that the dysregulation of specific miRNAs is linked to various asthma phenotypes, suggesting their potential as biomarkers for diagnosis and targets for treatment. MiRNAs like miR-126 play a role in regulating inflammatory cytokines and airway remodeling, thereby affecting the severity of asthma symptoms and exacerbations (22, 60). These molecules regulate inflammation, airway sensitivity, and tissue remodeling by interacting with specific mRNAs. Through these interactions, they influence the severity and progression of asthma. Among the ncRNAs that have received the most attention, miR-126 and miR-21 stand out as essential regulators of airway inflammation. Research has shown that miR-126 can amplify Th2 cell responses by boosting the production of IL-13 and eotaxin, which in turn worsens allergic inflammation in the airways. Interestingly, blocking miR-126 in animal models has been shown to lessen airway hyperreactivity and reduce eosinophilic infiltration, suggesting its potential as a therapeutic target (22). MiR-21, on the other hand, is implicated in glucocorticoid resistance and chronic inflammation. Kim et al. (2017) (61) demonstrated that miR-21 amplifies Phosphoinositide 3-kinase (PI3K) signaling and suppresses HDAC2, a regulator of corticosteroid sensitivity. These changes contribute to severe and treatment-refractory asthma phenotypes. These miRNAs play an important role as epigenetic regulators and could also serve as valuable biomarkers in diagnosing asthma and identifying its different types. This offers a minimally invasive approach for classifying patients.

LncRNAs have gained recognition as key regulators in many biological processes. They can control gene expression and affect immune responses, playing a role in the inflammatory pathways linked to asthma. Studies suggest that certain lncRNAs could be valuable as biomarkers or targets for asthma treatment, underscoring their involvement in the disease’s complexity. For example, lncZPBP2–3 has been linked to asthma susceptibility, with its expression being influenced by environmental factors such as particulate matter and polycyclic aromatic hydrocarbons (62). Lnc-Gm20716 plays a role in stress responses induced by HDM in bronchial epithelial cells (63). Reducing its expression can help ease these stress reactions through the Unc-51-like kinase 1(ULK1) signaling pathway (63). TP73-asthma1 worsens airway inflammation and remodeling by reducing the levels of let-7e 5′-arm microRNA (let-7e-5p), which may activate the High Mobility Group Box 1(HMGB1)/Receptor for Advanced Glycation End-products (RAGE)- Nuclear Factor kappa-light-chain-enhancer of activated B cells(NF-κB) pathway (64). A recent study found that 120 lncRNAs were upregulated and 1984 downregulated in C-X-C motif chemokine ligand 16(CXCL16) knockout mice, highlighting their potential as targets for regulating antigen presentation in asthma models (65). These findings highlight lncRNAs as potential therapeutic targets in asthma.

Key CpG sites have been identified that regulate Syntaxin 4 (STX4) expression, with higher methylation levels associated with a lower risk of childhood asthma, suggesting a protective epigenetic modification (66). DNA methylation patterns at birth and during childhood are associated with lung function development and asthma. In total, 1,049 differentially methylated CpGs have been identified, suggesting a role for epigenetic changes in asthma severity and lung function decline. Furthermore, changes in DNA methylation at genes related to respiration and immune function, caused by prenatal exposure to air pollutants, are linked to an increased asthma risk. Significant associations were identified across multiple CpG sites in genes relevant to asthma and allergy (67). In newborns, DNA methylation is associated with childhood asthma, with a newly identified differentially methylated position in Cell Adhesion Molecule 1 (CADM1) and 18 other regions. This suggests that DNA methylation may play a role in early asthma development, with sex-specific differences observed at certain genomic loci (68).

Histone modifications, including acetylation and methylation, influence chromatin structure and gene accessibility. Acetylation regulates gene expression by altering chromatin structure. This process can either promote or inhibit the transcription of genes involved in inflammation and immune responses in asthma. Metabolites like Acetyl coenzyme A (Acetyl-CoA) influence acetylation, directly linking metabolic processes to epigenetic regulation (69). In asthma models, treatments that reduce histone deacetylase 9 (HDAC9) expression, associated with increased short-chain fatty acid (SCFA) production and regulatory T Cells(Tregs) differentiation, suggest that acetylation processes may alleviate asthma symptoms (70). Additionally, N-acetyl-L-alanine, a metabolite linked to acetylation, is associated with an increased risk of developing obesity-related asthma (71). Histone modifications, like histone 3 lysine 4 mono-methylation (H3K4me1), have been associated with asthma development, suggesting they may play a role in worsening asthma symptoms (72). Additionally, ten-eleven translocation methylcytosine dioxygenase 1 (TET1) regulates gene expression in bronchial epithelial cells by modifying histones, particularly influencing histone 3 lysine 27 acetylation (H3K27ac) levels. This may help protect against changes in gene expression linked to asthma (73). The association between N6-methyladenosine (m6A) and H3K27ac in asthma highlights a complex regulatory network involving these epigenomic traits (74). In summary, the interaction between acetylation and histone modifications plays a key role in understanding the epigenetic mechanisms behind asthma, offering potential insights for therapeutic strategies. Epigenetic regulators offer both mechanistic insights into asthma heterogeneity and practical opportunities for precision intervention.

**Figure 10:**
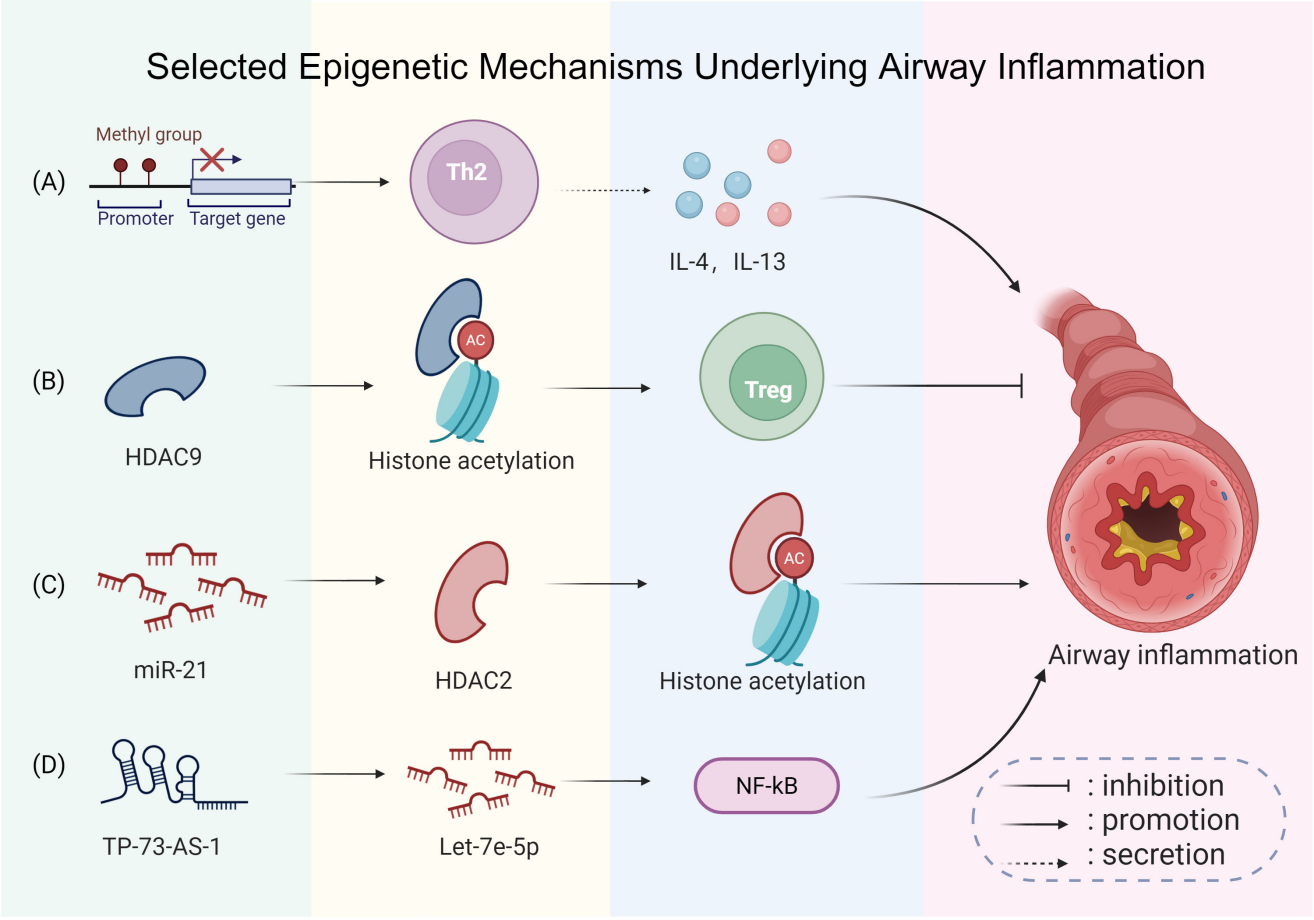
Selected epigenetic mechanisms underlying airway inflammation. **(A)** Site-specific DNA methylation changes are involved in asthma: alterations at certain immune-related loci can promote T-helper type 2 (Th2) polarization and enhance interleukin-4 (IL-4) and interleukin-13 (IL-13) expression, promoting airway inflammation. **(B)** Decreased expression of histone deacetylase 9 (HDAC9) is associated with enhanced Treg differentiation, which helps alleviate airway inflammation. **(C)** Conversely, upregulated microRNA-21 (miR-21) inhibits histone deacetylase 2 (HDAC2), resulting in exacerbated inflammation. **(D)** Furthermore, elevated tumor protein p73 antisense RNA 1 (TP73-AS1) downregulates let-7e 5′-arm microRNA (let-7e-5p), which may activate the high mobility group box 1 (HMGB1)/receptor for advanced glycation end-products (RAGE)/nuclear factor kappa-light-chain-enhancer of activated B cells (NF-κB) pathway, worsening airway inflammation and remodeling. This figure was created with BioRender.com and is used under license.

**Figure 10** summarizes some typical epigenetic mechanisms involved in airway inflammation, explaining how DNA methylation, histone deacetylase regulation, and non-coding RNAs interact to influence cytokine production and airway inflammation.

#### 4.1.3 Environmental exposures and risk factors

Chronic exposure to particulate matter less than 2.5 µm in diameter (PM2.5) greatly raises the risk of asthma symptoms in children, leading to impaired lung function and increased airway inflammation (75). Exposure to ozone is associated with a higher risk of coughing in children with asthma, with an odds ratio of 1.39, underscoring its importance as a major risk factor among air pollutants (76). Other environmental risk factors, including being overweight, a sedentary lifestyle, and exposure to environmental tobacco smoke, are strongly linked to asthma symptoms in young adolescents, highlighting the need for public health interventions (77). High particulate matter, nitrogen dioxide, and limited green space in urban areas are linked to a higher risk of asthma. An overall environmental risk score suggests that 11.6% of new asthma cases can be attributed to these exposures (78). In urban Indian adolescents, asymptomatic bronchial hyperresponsiveness (BHR) is associated with PM2.5 levels, indicating that these environmental factors could play a role in the early development of asthma (79). Prenatal ambient air pollution exposure is also associated with an increased risk of asthma, as indicated by the downregulation of the Axis inhibition protein 1(AXIN1) protein (80). Linking exposomic and epigenetic data offers a path to better risk prediction and targeted prevention in asthma.

#### 4.1.4 Epigenetic therapies and translational potential in asthma

Epigenetic therapies can modulate gene expression without altering DNA sequence, offering new avenues to address asthma’s underlying mechanisms.

New epigenetic therapies for asthma aim to modulate immune responses and enhance treatment effectiveness. Targeting reactive oxygen species (ROS) to regulate the polarization of classically activated (M1) and alternatively activated (M2) macrophages shows promise in restoring immune balance, which may help alleviate airway hyperresponsiveness in patients with asthma (81). These advancements highlight the potential of epigenetic interventions in developing more effective asthma therapies.

A variety of biomarkers—such as specific proteins, inflammatory mediators, and genetic variants—are used to identify asthma phenotypes, predict exacerbation risk, and monitor treatment responses. Incorporating epigenetic markers into clinical practice allows physicians to track disease progression more precisely, anticipate exacerbations, and tailor treatment plans. This approach may lead to better long-term asthma control. For instance, Stikker et al. (2023) (82) reported that methylation at loci near ORMDL sphingolipid biosynthesis regulator 3 (ORMDL3) and Gasdermin B (GSDMB)—two genes on the 17q12–21 asthma susceptibility locus—was significantly associated with early-onset asthma and more frequent exacerbations. These epigenetic signatures also correlated strongly with exposure to airborne pollutants and viral infections, suggesting their value in mapping environment–gene interactions. Taken together, these findings emphasize how epigenetic mechanisms not only regulate key immunological and inflammatory pathways in asthma, but also offer novel targets for early detection, phenotypic classification, and precision therapy.

In light of this, there is hope for the development of precise therapies to regulate immune responses and airway inflammation. MiRNAs, in particular, have become important therapeutic targets in asthma, especially in regulating immune and epithelial cell functions. For example, antagomirs targeting miR-21, which promotes eosinophilic inflammation and steroid resistance, have been found to reduce airway hyperresponsiveness and lower IL-13 levels in mouse models (83). Additionally, microRNA-146a(miR-146a), a negative regulator of NF-κB signaling, has been proposed as a potential anti-inflammatory target, especially for neutrophilic asthma phenotypes (84). Although clinical trials of miRNA-based therapies for asthma are still limited, their successful use in other diseases, such as miravirsen for Hepatitis C Virus (HCV) infection, highlights their potential and therapeutic promise. Overall, integrating epigenetic therapies with existing treatment paradigms may change long-term disease management.

### 4.2 Prospects for the future

Future research in asthma epigenetics will focus on understanding the complex gene-environment interactions, especially the roles of DNA methylation, histone modifications, and miRNAs in immune regulation and corticosteroid response. A major goal is to translate epigenetic biomarkers into clinical practice, enabling precision medicine through phenotype-specific diagnostics and treatments. Epigenetic therapies—like HDACis and miRNA-based drugs—offer promising targeted interventions. Additionally, further research into early-life environmental exposures and their long-term epigenetic effects is crucial for developing effective prevention strategies. Collaboration across disciplines and improved data sharing will be key to advancing personalized, mechanistically driven asthma management.

#### 4.2.1 Clinical translation: biomarkers and patient stratification

Several clinical studies have begun to identify epigenetic biomarkers that correlate with asthma severity, exacerbation frequency, and treatment response, thereby supporting their use in patient stratification and personalized treatment plans. A study by Xue Zhang et al. (2017) (85) profiled nasal epithelial DNA methylation in children and identified signatures predictive of asthma control and exacerbation risk. Similarly, methylation at TET1 and FOXP3 loci has been associated with corticosteroid sensitivity, providing a basis for tailored therapy decisions (86, 87). In parallel, biologic therapies such as monoclonal antibodies against IgE and interleukin-5 (IL-5) have also proven effective in reducing exacerbation frequency and improving lung function, underscoring the broader move toward precision medicine in severe asthma (88). These findings suggest that integrating epigenetic biomarkers into patient stratification frameworks could transform asthma management by enabling earlier intervention and individualized treatment strategies.

#### 4.2.2 Advances in epigenetic therapies for asthma

In recent years, epigenetic-modifying drugs originally developed for cancer and autoimmune diseases have begun to be explored for respiratory conditions such as asthma. Among these, histone deacetylases (HDACs) have received particular attention. HDAC2, which regulates glucocorticoid receptor function and suppresses inflammatory gene expression, is often reduced in patients with steroid-resistant asthma (9). Preclinical studies demonstrate that low-dose theophylline can restore HDAC activity in inflammatory airway cells. For instance, theophylline increased HDAC activity in alveolar macrophages from patients with chronic obstructive pulmonary disease, thereby enhancing corticosteroid-mediated anti-inflammatory effects (89). While the exact mechanisms remain to be fully clarified, these findings support the therapeutic potential of targeting HDAC2 in overcoming corticosteroid resistance.

Moreover, researchers have begun investigating DNA methyltransferase inhibitors (DNMTis) like 5-azacytidine in animal models, aiming to reverse the methylation of anti-inflammatory genes that have been silenced. Although these approaches remain primarily within the realm of preclinical studies, they hold significant promise in reshaping immune responses in the airways, particularly in cases where environmental factors and early-life epigenetic changes contribute to the development of certain phenotypes.

Epigenetic therapies for asthma have highlighted the role of genetic variations in the histamine N-methyltransferase (HNMT) gene, which are associated with allergic asthma. Studies have shown that specific genotypes, such as HNMT-464 TT and HNMT-1639 TT, are overrepresented in allergic children compared to nonallergic children, suggesting a genetic predisposition linked to these polymorphisms (90). Additionally, the TT genotype and T allele of the Thr105Ile polymorphism in the HNMT gene have been associated with an increased risk of asthma in children, indicating that these functional polymorphisms may modify susceptibility to the condition (91). These findings underscore the potential for targeted epigenetic therapies that address specific genetic variations in the HNMT gene, offering a promising avenue for personalized treatment strategies in managing asthma. Together, these advances highlight epigenetic modulation as a powerful approach to overcome steroid resistance and guide mechanism-driven precision therapies.

### 4.3 Limitations

This study presents a comprehensive bibliometric analysis of asthma research in the field of epigenetics, covering the period from 1980 to 2025. Using tools such as VOSviewer, CiteSpace, and the bibliometrix R package, we visualized collaboration networks, pinpointed key countries, institutions, and authors, and identified key research themes like “DNA methylation,” “miRNA regulation,” and “environmental exposures.” Employing a variety of analytical methods and metrics—such as the h-index, g-index, and citation bursts—offered a multi-faceted view of the research landscape. With 4,020 publications and 221,863 references, the study ensures extensive coverage and provides a robust analysis of trends over the past forty-five years.

Despite its strengths, this study has three key limitations. First, our reliance on the WoSCC and Scopus may have led to omissions or inconsistencies due to differing indexing criteria and journal coverage. Second, limiting our analysis to English-language publications might underrepresent valuable contributions from regions like China, Russia, and Latin America. Third, the focus on quantitative metrics—such as publication and citation counts—may overlook the qualitative significance or innovation of influential but less-cited works. To overcome these challenges, future studies should broaden their database selection including CNKI and other regional indices, embrace multilingual literature, and consider incorporating alternative impact measures, such as peer reviews or expert surveys, to capture a more complete picture of scholarly influence.

## 5 Conclusion

Epigenetic research is reshaping asthma treatment by advancing personalized and molecularly targeted approaches. Studies on DNA methylation, histone modifications, and non-coding RNAs have revealed key mechanisms underlying airway inflammation, supporting patient stratification and biomarker discovery. Novel interventions such as histone deacetylase inhibitors, DNA methyltransferase inhibitors, and microRNA-based therapies show potential to restore immune balance and enhance corticosteroid responsiveness, although most remain in early clinical phases. Critical challenges—including reliable biomarker validation, effective drug delivery, and long-term outcome evaluation—must be resolved before translation into clinical practice. With continued progress, epigenetic strategies may enable a paradigm shift from symptom management toward precision therapies, ultimately improving outcomes and long-term disease control in asthma.

## Data Availability

The raw datasets analyzed in this study were obtained from Web of Science Core Collection and Scopus via institutional subscriptions and are not publicly available due to copyright restrictions. Processed data (e.g., extracted keywords, citation networks) are available upon reasonable request. Requests to access these datasets should be directed to BZ, 202500220279@sxmu.edu.cn.

## Glossary

AXIN1: Axis inhibition protein 1
BHR: bronchial hyperresponsiveness
CA-NPs-Quercetin: Caffeic acid–loaded nanoparticles with Quercetin
CADM1: Cell Adhesion Molecule 1
CpG: Cytosine–phosphate–Guanine dinucleotide
CXCL16: C-X-C motif chemokine ligand 16
DNMTis: DNA methyltransferase inhibitors
FCN-A: Ficolin-A
FOXP3: Forkhead box P3
GSDMB: Gasdermin B
H3K27ac: Histone 3 lysine 27 acetylation
H3K4me1: Histone 3 lysine 4 mono-methylation
HCV: Hepatitis C Virus
HDAC2: Histone deacetylase 2
HDAC9: Histone deacetylase 9
HDACis: Histone deacetylase inhibitors
HDACs: Histone deacetylases
HDM: House dust mite
HMGB1: High Mobility Group Box 1
HNMT: N-methyltransferase
IgE: Immunoglobulin E
IL-4: Interleukin-4
IL-5: Interleukin-5
IL-10: Interleukin-10
IL-13: Interleukin-13
ILC2s: Group 2 innate lymphoid cells
let-7e-5p: let-7e 5′-arm microRNA
lncRNA: Long non-coding RNA
M-CSF: Macrophage colony-stimulating factor
m6A: N6-methyladenosine
M1: Classically activated macrophages
M2: Alternatively activated macrophages
miRNA: microRNA
miR-21: microRNA-21
miR-126: microRNA-126
miR-146a: microRNA-146a
miR-155: microRNA-155
ncRNA: Non-coding RNA
NF-κB: Nuclear Factor kappa-light-chain-enhancer of activated B cells
ORMDL3: ORMDL sphingolipid biosynthesis regulator 3
OVA: Ovalbumin
PI3K: Phosphoinositide 3-kinase
PM2.5: Particulate matter less than 2.5 µm in diameter
RAGE: Receptor for Advanced Glycation End-products
ROS: Reactive oxygen species
SCFA: Short-chain fatty acid
STING: Stimulator of Interferon Genes
STX4: Syntaxin 4
TET1: Ten-eleven translocation methylcytosine dioxygenase 1
Th2: T helper type 2 cells
Th17: T helper 17 cells
TLRs: Toll-like receptors
TP73-AS1: Tumor protein p73 antisense RNA 1
Tregs: Regulatory T cells
TSA: Trichostatin A
ULK1: Unc-51-like kinase 1
WoSCC: Web of Science Core Collection.
