# Supplementary material for: Immune and inflammatory mechanisms in asthma: insights into epigenetic modifications
Source: Front Immunol. 2025 Oct 8;16:1677552. doi: 10.3389/fimmu.2025.1677552 (PMC12540441; doi:10.3389/fimmu.2025.1677552)
Supplement: Supplementary file 1 [file Table1.docx]

**Supplementary Table S1. Bibliometric Overview of Asthma in Epigenetics Field (1980–2025)**

| **Description** | **Results** |
| --- | --- |
| MAIN INFORMATION ABOUT DATA |  |
| Timespan | 1980:2025 |
| Sources (Journals, Books, etc) | 1104 |
| Documents | 4020 |
| Annual Growth Rate % | 11.98 |
| Document Average Age | 7.54 |
| Average citations per doc | 53.47 |
| References | 221863 |
| DOCUMENT CONTENTS |  |
| Keywords Plus (ID) | 21791 |
| Author's Keywords (DE) | 6909 |
| AUTHORS |  |
| Authors | 20197 |
| Authors of single-authored docs | 202 |
| AUTHORS COLLABORATION |  |
| Single-authored docs | 242 |
| Co-Authors per Doc | 7.14 |
| International co-authorships % | 14.73 |
| DOCUMENT TYPES |  |
| article | 3360 |
| review | 660 |
