# Supplementary material for: Immune and inflammatory mechanisms in asthma: insights into epigenetic modifications
Source: Front Immunol. 2025 Oct 8;16:1677552. doi: 10.3389/fimmu.2025.1677552 (PMC12540441; doi:10.3389/fimmu.2025.1677552)
Supplement: Supplementary file 2 [file Table2.docx]

**Supplementary Table S2. Most Relevant Countries by Corresponding Author Contributions**

| Rank | **Country** | **Articles** | **Articles %** | **SCP** | **MCP** | **MCP %** |
| --- | --- | --- | --- | --- | --- | --- |
| 1 | CHINA | 681 | 16.9 | 625 | 56 | 8.2 |
| 2 | USA | 567 | 14.1 | 426 | 141 | 24.9 |
| 3 | UNITED KINGDOM | 144 | 3.6 | 95 | 49 | 34 |
| 4 | KOREA | 103 | 2.6 | 93 | 10 | 9.7 |
| 5 | AUSTRALIA | 93 | 2.3 | 52 | 41 | 44.1 |
| 6 | GERMANY | 86 | 2.1 | 43 | 43 | 50 |
| 7 | CANADA | 65 | 1.6 | 45 | 20 | 30.8 |
| 8 | SPAIN | 61 | 1.5 | 38 | 23 | 37.7 |
| 9 | INDIA | 47 | 1.2 | 37 | 10 | 21.3 |
| 10 | ITALY | 47 | 1.2 | 35 | 12 | 25.5 |
| 11 | JAPAN | 45 | 1.1 | 39 | 6 | 13.3 |
| 12 | NETHERLANDS | 41 | 1 | 16 | 25 | 61 |
| 13 | SWEDEN | 35 | 0.9 | 14 | 21 | 60 |
| 14 | IRAN | 33 | 0.8 | 25 | 8 | 24.2 |
| 15 | POLAND | 31 | 0.8 | 27 | 4 | 12.9 |
| 16 | SWITZERLAND | 27 | 0.7 | 3 | 24 | 88.9 |
| 17 | FRANCE | 21 | 0.5 | 10 | 11 | 52.4 |
| 18 | BRAZIL | 17 | 0.4 | 12 | 5 | 29.4 |
| 19 | BELGIUM | 16 | 0.4 | 8 | 8 | 50 |
| 20 | EGYPT | 16 | 0.4 | 11 | 5 | 31.3 |
| 21 | NORWAY | 15 | 0.4 | 5 | 10 | 66.7 |
| 22 | GREECE | 14 | 0.3 | 9 | 5 | 35.7 |
| 23 | MEXICO | 11 | 0.3 | 7 | 4 | 36.4 |
| 24 | DENMARK | 10 | 0.2 | 5 | 5 | 50 |
| 25 | FINLAND | 9 | 0.2 | 2 | 7 | 77.8 |

Abbreviation: SCP: Single Country Publications; MCP: Multiple Country Publications
